# From magic spot ppGpp to MESH1: Stringent response from bacteria to metazoa

**DOI:** 10.1371/journal.ppat.1011105

**Published:** 2023-02-02

**Authors:** Jen-Tsan Chi, Pei Zhou

**Affiliations:** 1 Department of Molecular Genetics and Microbiology, Duke University School of Medicine, Durham, North Carolina, United States of America; 2 Duke Center for Genomic and Computation Biology, Duke University School of Medicine, Durham, North Carolina, United States of America; 3 Department of Biochemistry, Duke University School of Medicine, Durham, North Carolina, United States of America; University of Geneva: Universite de Geneve, SWITZERLAND

## 1 What are the magic spot and bacterial stringent response?

All organisms are constantly exposed to varying levels of nutrients and environmental stresses. This is especially true for unicellular organisms, such as bacteria, which are exposed to perpetual changes of outside environments. Therefore, it is critical to have mechanisms to sense, adapt, and cope with various nutrient deprivations and metabolic stresses. In bacteria, one of the major stress adaptive responses is the “stringent response” [1] that enables bacteria to transition into a semi-dormant state characterized by proliferation arrest, stress survival, and metabolic/transcriptome reprogramming. The stringent response is triggered by the alarmone (p)ppGpp, also termed the magic spot which dramatically increases it level under stresses and binds to various protein targets to mediate the stringent response [1]. The (p)ppGpp level is regulated by the balance of its synthesis and degradation (hydrolysis) by the proteins in the RelA/SpoT homologues (RSHs) superfamily [2]. The best-characterized RSH proteins are the multi-domain long RHS proteins involved in the (p)ppGpp synthesis (RelA) and hydrolysis (SpoT). In addition, there are also single-domain RSH proteins known as small alarmone synthetases (SASs) or small alarmone hydrolases (SAHs) mediating the synthesis and hydrolysis of alarmones, respectively [3]. Interestingly, SAHs have been classified into multiple subgroups including the Mesh1 and Mesh1-L subfamilies [2]. However, the physiological function of these short RSH proteins is still not completely understood.

## 2 How do magic spot and alarmone mediate stringent response?

**Fig 1:**
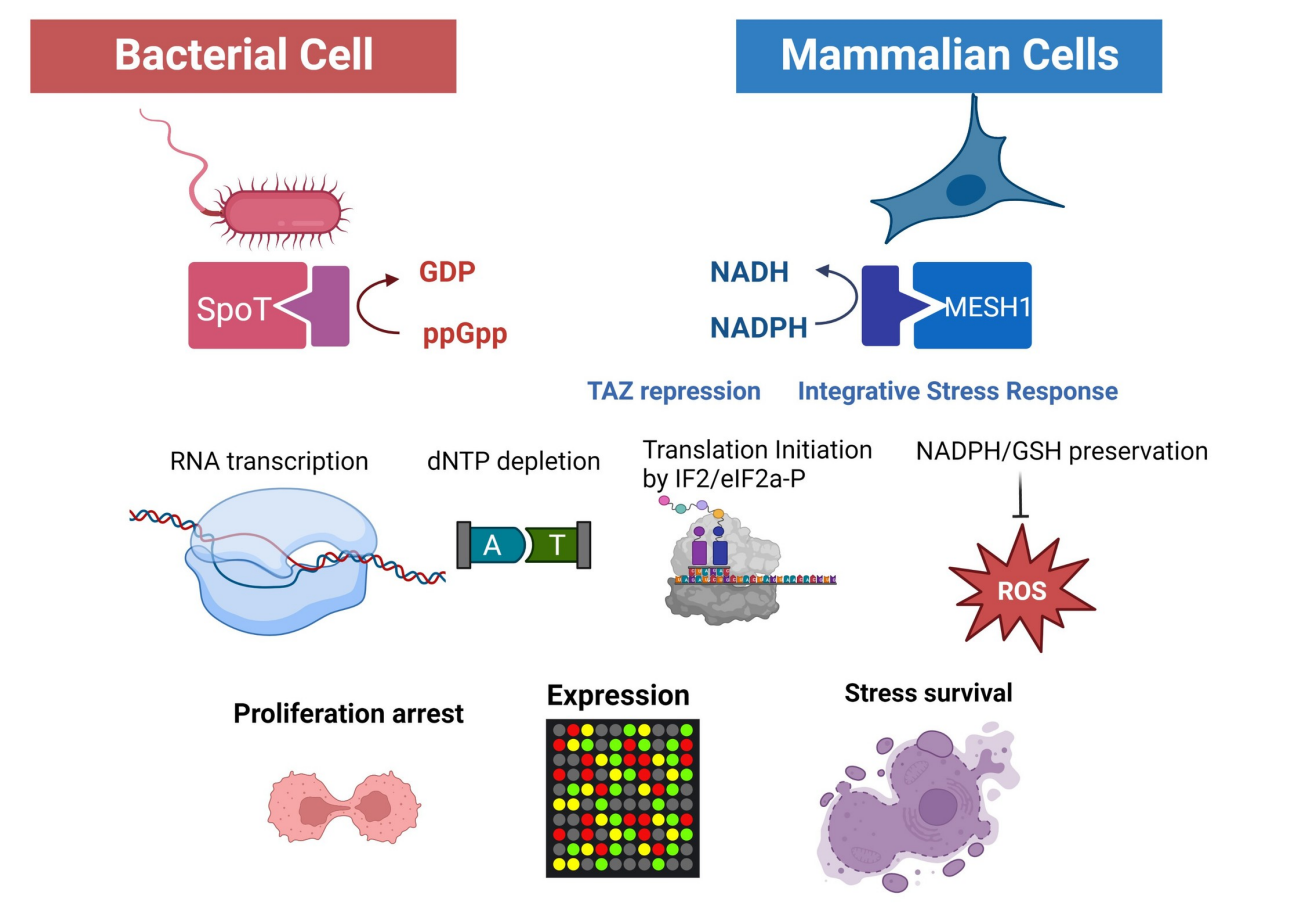
Distinct substrates of SpoT/MESH1 and the conserved biological processes and phenotypic features of the bacterial (left) and mammalian (right) stringent response.

The stringent response involves the coordinated alterations in the bacterial transcription, physiology, and metabolisms. During stringent response, (p)ppGpp plays a central role in this wide variety of changes by binding to its many intracellular protein and RNA targets. While some targets are common across bacteria, other targets are only relevant to particular species and life style stages. The extensive discussion of the diverse (p)ppGpp targets, affected biological processes, and heterogeneity among bacteria has been covered in several outstanding reviews [1,4,5]. While there are common and conserved themes, the specific protein targets and regulatory mechanisms of (p)ppGpp may differ between different bacterial species. For example, while (p)ppGpp affects the global and regional transcription of *Escherichia coli* through the direct binding to the DksA and the RNA polymerase (RNAP) [6], it affects transcription by affecting the GTP level and binding to specific transcription factors, such as PurR [7] and MglA/SspA complex [8], in other bacteria. In addition, (p)ppGpp can reduce DNA proliferation by binding to DnaG (synthetase of the priming RNA required for DNA replication) [9], affecting the expression and stability of DnaA (replication initiation ATPase) [10] and modulating the supercoiling state of oriC [11]. Furthermore, (p)ppGpp reduces cellular nucleotides through substrate depletion and inhibition of multiple enzymes (PurF, GuaB, and Gmk) mediating the nucleotide biosynthesis [12–14]. At the level of protein translation, (p)ppGpp reduces the translation initiation through binding to the initiation factor IF2 [15] and inhibiting ribosomal assembly [16,17]. Therefore, (p)ppGpp binds to numerous protein targets in different biological processes to mediate a wide variety of phenotypic changes in the bacterial stringent response (Fig 1).

## 3 MESH1 is the SpoT homolog in metazoan genomes

For many years, (p)ppGpp and the stringent response was thought to be only relevant in bacteria and plants, but not in metazoa [18]. Interestingly, metazoan genomes contain MESH1—Metazoan SpoT Homolog 1, encoded by *HDDC3* (HD Domain Containing 3), even though no similar homolog of RelA is found in metazoa. MESH1 can also hydrolyze (p)ppGpp, suggesting a conservation of the biochemical function between MESH1 and SpoT [19]. However, (p)ppGpp was found to exist at a very low level in metazoan (approximately 10^−6^-fold lower than in bacteria) [20]. Therefore, the function and relevant substrate of MESH1 remained a mystery for a long time. Interestingly, the removal of *Mesh1* from *Drosophila* triggered a transcriptome response reminiscent of the bacterial stringent response [19], suggesting a functional conservation across evolution.

## 4 MESH1 removal robustly protected ferroptosis of cancer cells

The function of MESH1 in human was uncovered during a forward genetic screen of ferroptosis, a newly recognized form of stress-induced cell death characterized by oxidative stress, iron dependence, and lipid peroxidation [21,22]. While first discovered during the investigation of the cell-killing ability of erastin [21], ferroptosis is now appreciated to be broadly relevant in multiple settings, including tumor suppression, neurodegeneration, liver cirrhosis, and ischemia-reperfusion injuries [22,23]. In addition, ferroptosis also plays a role in the host–pathogen interactions [24]. To identify genetic determinants of ferroptosis triggered by cystine deprivation, we performed several forward genetic screens [25,26] and identified MESH1 as a top hit as its knockdown robustly protected ferroptosis of all tested cells for up to 1 week [27]. Therefore, MESH1 is a novel and robust regulator of ferroptosis of human cancer cells.

## 5 Additional phenotypic analysis of MESH1 knockdown–phenotypic conservations

In addition to the ferroptosis survival, MESH1 knockdown also triggers additional phenotypic responses. First, there is a robust proliferation arrest in all tested cancer cell lines, tumor spheres, and xenografts in mice (Fig 1). Transcriptome analysis revealed a significant reduction of dNTP synthesis genes and depletion of dNTPs [28], the building blocks of DNA synthesis. In addition, there is a depletion of the ribosomal gene set [29], implying a reduction in the expression of ribosome-associated genes, similar to what has been described in the bacterial stringent response (Fig 1). Among the affected genes is the prominent repression of TAZ, but not YAP, two co-activators of TEAD transcriptional factors downstream of the Hippo pathways. It is important to note that YAP/TAZ proteins are often co-regulated by protein modification and subcellular translocation. Therefore, the selective transcription repression of TAZ mRNAs upon MESH1 knockdown is a novel mechanism that occurs by histone hypoacetylation. Importantly, TAZ restoration significantly mitigates the many phenotypic changes induced by MESH1 knockdown, including the proliferation arrest and dNTP depletion, highlighting the important role of TAZ repression [28]. The integrated stress response (ISR) is an evolutionarily conserved intracellular signaling network that is activated upon stresses to maintain homeostasis and survival [30]. Interestingly, MESH1 knockdown induced integrative stress responses (ISR) as evidenced by the increased levels of *ATF4* protein and eIF2α phosphorylation and induction of *ATF3*, *XBPs*, and *CHOP* mRNA [29]. Similar to our observation in human cells, (p)ppGpp has been shown to bind to IF2a and inhibit translation initiation [15] (Fig 1). Another study of MESH1 in *Caenorhabditis elegans* also reported the induction of unfolding protein response upon MESH1 removal [31]. Therefore, there are significant similarities between the bacterial and metazoan stringent response across evolution, prompting us to coin the term of “metazoan stringent-like response” [32].

## 6 The relevant substrate of MESH1 is revealed

**Fig 2:**
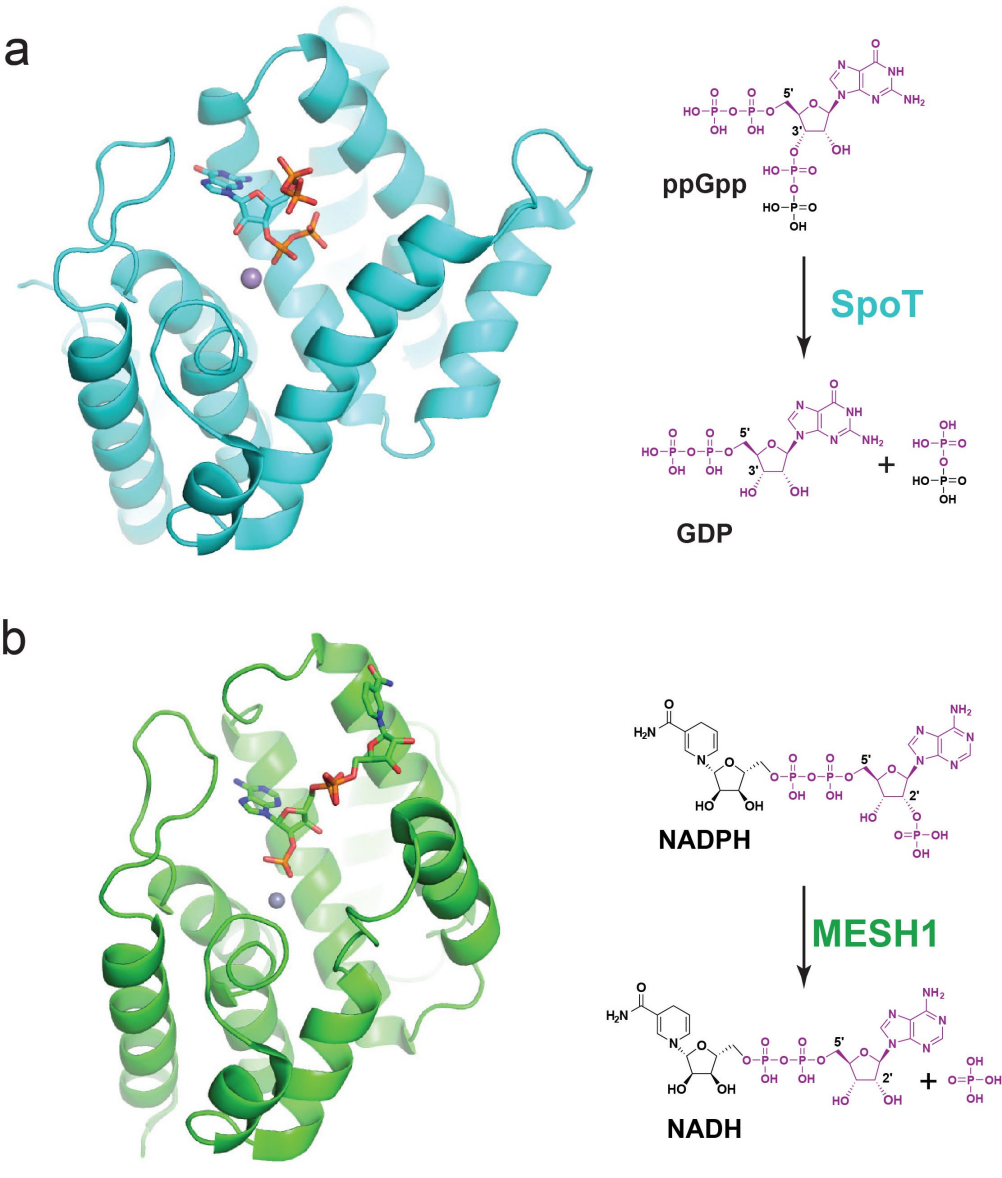
Comparison between Rel/SpoT and MESH1. (a) Crystal structure of *Thermus thermophilus* Rel in complex with ppGpp (PDB: 6S2T). The hydrolase domain of Rel/SpoT hydrolyzes ppGpp to yield GDP and pyrophosphate. (b) Crystal structure of MESH1 in complex with NADPH (PDB: 5VXA). MESH1 hydrolyzes NADPH to yield NADH and phosphate. Rel and MESH1 are shown in the cartoon model, ppGpp and NADPH in the stick model, and active site Mn^2+^ ions shown in the sphere model.

While *MESH1* knockdown triggers a strong phenotypic response, it is not clear what the relevant substrate(s) are in human cells. We discovered that MESH1 is a NADPH phosphatase capable of removing the 2′-phosphate of NADPH to form NADH [27] (Fig 2). While both NADPH and (p)ppGpp contain a purine moiety, the enzymatic activity of MESH1 toward NADPH is the cleavage at the 2′-position of ribose, in contrast to the 3′-position in (p)ppGpp. The catalytic efficiency (*k*_cat_/*K*_M_) toward NADPH (14.4 × 10^3^ M^−1^ s^−1^) [27] is similar to the reported enzymatic activities toward (p)ppGpp (9.46 × 10^3^ M^−1^ s^−1^) [19]. Furthermore, the binding mode of NADPH was captured through the crystal structure of the catalytic inactive MESH1 D66K mutant in complex with NADPH (PDB: 5VXA), providing detailed molecular interactions between MESH1 and NADPH (Fig 2). The NADPH phosphatase activity of MESH1 has been validated in an independent study using the *C*. *elegans* homologue of MESH1 [31], verifying NADPH as the relevant metazoan substrate for MESH1. Interestingly, a recent paper from Wang lab has confirmed the NADPH phosphatase activities of SAH in phytopathogen *Xanthomonas campestris* pv. *campestris* (*Xcc*) and *sah* loss strongly reduced NADH, showing the evolutionary conservation of the NADPH phosphatase activities across multiple kingdoms [33].

## 7 Implication for our understanding of stringent response and remaining questions

The stringent response is an ancient and evolutionarily conserved stress response to enable the stress survival of bacteria, providing the primary means by which bacteria survive metabolic stresses. Upon stress relief, the drop in (p)ppGpp ensures the rapid resolution of stringent response to resume normal proliferative states. Similarly, MESH1 knockdown in cancer cells also triggers similar sets of biological processes as well as the phenotypic features of stress survival, proliferation arrests, and transcriptional reprogramming that are highly similar to bacterial stringent response (Fig 1). Therefore, we termed these phenotypic responses to MESH1 inhibition as metazoan stringent-like response [32]. However, while (p)ppGpp binds and modulates various molecular targets in bacteria, several additional pathways (such as TAZ, integrative stress response) connect MESH1 to downstream biological processes (Fig 1). In addition, there is also an evolutionary acquisition of distinct substrates of SpoT versus MESH1. During evolution, enzymes may have altered substrate specificity for a new substrate or broader substrate specificity, as shown for the ability of *Xcc* SAH to hydrolyze both (p)ppGpp and NADPH [33]. Evolutionary changes in substrate preferences can coincide with the development of new signaling pathways. Despite these advances, much remains unknown about MESH1 and the metazoan stringent-like response. It is unknown what stresses and external stimuli could induce the stringent response in metazoa. Furthermore, it is not clear what the phenotypic response of the MESH1 removal is at the organism level in animals, as reported in *C*. *elegans* [31]. MESH1 appears to play a significant role in tumor biology and the inhibition of MESH1 by chemical inhibitors could be used to trigger mammalian stringent-like response to treat various cancers. The field of the metazoan stringent response is in its infancy, and much is to be explored about this important signaling system and its similarity and difference from the bacterial stringent response.
